# Preparation of Chitosan–Hexaconazole Nanoparticles as Fungicide Nanodelivery System for Combating *Ganoderma* Disease in Oil Palm

**DOI:** 10.3390/molecules24132498

**Published:** 2019-07-08

**Authors:** Farhatun Najat Maluin, Mohd Zobir Hussein, Nor Azah Yusof, Sharida Fakurazi, Abu Seman Idris, Nur Hailini Zainol Hilmi, Leona Daniela Jeffery Daim

**Affiliations:** 1 Institute of Advanced Technology, Universiti Putra Malaysia, Serdang 43400, Malaysia; 2 Department of Chemistry, Faculty of Science, Universiti Putra Malaysia, Serdang 43400, Malaysia; 3 Department of Human Anatomy, Faculty of Medicine and Health Sciences, Universiti Putra Malaysia, Serdang 43400, Malaysia; 4 Malaysian Palm Oil Board (MPOB), 6, Persiaran Institusi, Bandar Baru Bangi, Kajang 43000, Malaysia; 5 Sime Darby Technology Centre Sdn. Bhd., UPM-MTDC Technology Centre III, Lebuh Silikon, Universiti Putra Malaysia, Serdang 43400, Malaysia

**Keywords:** agronanoparticles, nanodelivery system, fungicide, antifungal, *Ganoderma boninense*

## Abstract

Fungicide is used to control fungal disease by destroying and inhibiting the fungus or fungal spores that cause the disease. However, failure to deliver fungicide to the disease region leads to ineffectiveness in the disease control. Hence, in the present study, nanotechnology has enabled the fungicide active agents (hexaconazole) to be encapsulated into chitosan nanoparticles with the aim of developing a fungicide nanodelivery system that can transport them more effectively to the target cells (*Ganoderma* fungus). A pathogenic fungus, *Ganoderma boninense* (*G. boninense*), is destructive to oil palm whereby it can cause significant loss to oil palm plantations located in the Southeast Asian countries, especially Malaysia and Indonesia. In regard to this matter, a series of chitosan nanoparticles loaded with the fungicide, hexaconazole, was prepared using various concentrations of crosslinking agent sodium tripolyphosphate (TPP). The resulting particle size revealed that the increase of the TPP concentration produced smaller particles. In addition, the *in vitro* fungicide released at pH 5.5 demonstrated that the fungicide from the nanoparticles was released in a sustainable manner with a prolonged release time up to 86 h. On another note, the *in vitro* antifungal studies established that smaller particle size leads to lower half maximum effective concentration (EC_50_) value, which indicates higher antifungal activity against *G. boninense*.

## 1. Introduction

The application of nanotechnology in agriculture has attracted a considerable amount of attention [1,2,3] because it facilitates a better delivery system for agricultural chemicals which include fertilizers, pesticides, herbicides, fungicides, plant growth regulators, and others [4,5,6,7]. Nanoscale carriers offer several advantages including a more capable delivery system, productive storage, and controlled release properties [8,9] through encapsulation and entrapment, polymers, and surface ionic and weak bond attachments. In addition, it improves the stability which is believed to be helpful in preventing environmental degradation, thereby reducing chemical overflow and ecological issues [10,11]. Meanwhile, their controlled release properties are capable of restricting the amount of active ingredient, which consequently lessens the agricultural chemical waste and minimizes pollution [12,13,14,15]. Furthermore, these nanoscale carriers can be equipped with the ability to attach to plant roots or the surrounding soil structure and organic matter through the use of molecular and conformational affinity on the delivery nanoscale structure as well as matters in the soil [16,17,18].

*Ganoderma* disease resulting from the fungus *Ganoderma boninense (G. boninense)* is regarded as one of the critical issues in oil palm cultivation [19,20,21,22]. *G. boninense* can only be detected when the oil palm is internally infected by about 50%, thus making it impossible for early control and treatment, resulting in reduced palm oil output [20]. In addition, the fungus releases its spores and then forms on the exterior of the trunk which causes the disease to be easily spread on the soil or nearby trees [21,23]. As a result, *Ganoderma* has yielded a significant loss to the oil palm industry [24].

In regard to this issue, it is worth acknowledging that chitosan is the second richest polysaccharide which can easily be attained in nature. They can be found in different types of entities except in higher plants and vertebrate animals. In plant protection, chitosan is reported to be used as a seed treatment and as a fungicide nanocarrier in helping plants to fight off fungal infections. Basically, chitosan is reported to be able to enhance plant defense mechanisms and control or reduce the spreading of disease in the plant by inhibiting the fungus pathogens [25,26,27,28,29]. More importantly, chitosan shows several advantages which include nontoxicity, biodegradability, biocompatibility, antimicrobial activity, and antioxidant activity. On another note, tripolyphosphate (TPP), which acts as a crosslinking agent, is added in the preparation of chitosan nanoparticles (ionic gelation method) [30,31] considering that it is toxin-free and a multivalent anion. Moreover, the cationic chitosan can interact with the anionic TPP using electronic forces. Apart from that, TPP has been reported to be capable of controlling the nanoparticles size and drug loading [31,32].

Moreover, there are several previous works reported in the development of fungicide delivery systems using polymers, β-cyclodextrin, silica, and chitosan as the carrier system [4]. Owing to the easy formulation and ability to encapsulate both hydrophilic and hydrophobic fungicide, chitosan nanoparticles are shown to be a promising candidate as the nanocarrier [33].

Recent work has established that fungicidal treatments of hexaconazole are definitely capable of treating *Ganoderma* disease in oil palm [34,35]. In regard to this matter, it should be noted that hexaconazole is classified under the triazole group, which is competent in extending the fertility of *Ganoderma*-infected palms in comparison to other typical fungicides such as thiram, benomyl, triadimefon, triadimenol, and tridemorph. In addition, triazole fungicides, particularly Ascomycetes and Basidiomycetes [36], have been extensively applied on cereals and ornamental plants due to their ability in restricting the growth of fungi. Hexaconazole is represented as (*RS*)-2-(2,4-dichlorophenyl)-1-(1*H*-1,2,4-triazole-1-yl)hexane-2-ol and consists of systemic demethylation inhibitors that primarily work on the vegetative stage of fungi which hinders the mycelial development either inside or on the surface of the host plant [37].

The aim of the present study was to develop a fungicide nanodelivery formulation with slow release capability, low toxicity, and high antifungal activity towards *G. boninense* by encapsulation of hexaconazole into the chitosan nanocarrier. In the present study, the nanoparticles of chitosan–hexaconazole were optimized first by varying the concentration of sodium tripolyphosphate (TPP) to control the particle size distribution, loading content, and encapsulation efficiency of hexaconazole with the overall aim of controlling *Ganoderma* disease in oil palm. Apart from that, the current research also aimed to investigate the effect of size of chitosan–hexaconazole nanoparticles on *in vitro* antifungal activity against *G. boninense* together with the release behavior of hexaconazole.

## 2. Results and Discussions

### 2.1. Nanoparticle Characterizations

#### 2.1.1. Reaction Yield, Hexaconazole Loading Content, and Encapsulation Efficiency

As listed in Table 1, the reaction yield in the preparation of chitosan–hexaconazole nanoparticles reached optimum at 5 mg/mL of TPP. No significant difference was observed in the increase in TPP concentrations. Likewise, loading content (LC) and encapsulation efficiency (EE) were seen to reach saturation at 5 mg/mL of TPP, where the increase of TPP concentrations did not increase the LC and EE value anymore. These might be due to the smaller particle size in the synthesized nanoparticles at 10 and 20 mg/mL of TPP, which will be discussed later.

#### 2.1.2. Powder X-Ray Diffraction

**Figure 1:**
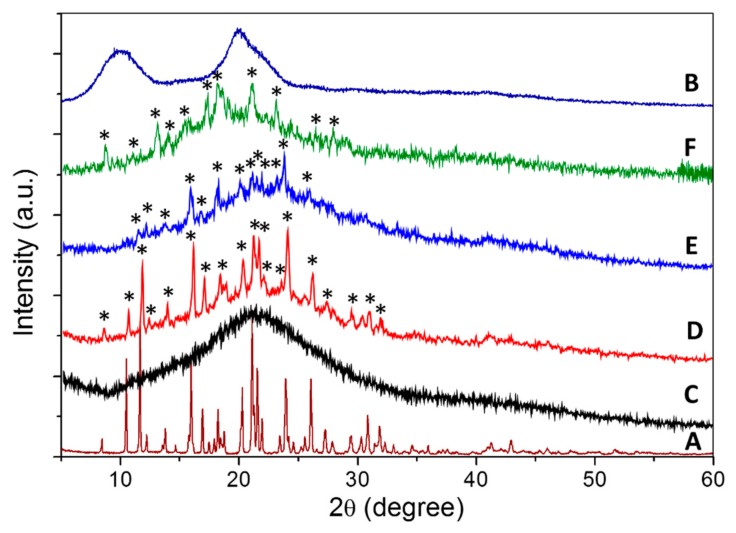
Powder XRD patterns of (**A**) pure hexaconazole, (**B**) CEN and chitosan–hexaconazole nanoparticles prepared at various concentrations of TPP, (**C**) 2.5, (**D**) 5, (**E**) 10, and (**F**) 20 mg/mL. The asterisk represents the hexaconazole peaks.

As shown in Figure 1, CEN showed a broad peak showing they are amorphous in nature, while pure hexaconazole showed a sharp peak, suggesting that they are a highly crystalline material. For the synthesized nanoparticles, CHEN2.5 showed only the broad peak of amorphous chitosan, while in CHEN5, CHEN10, and CHEN20, the crystalline peak of hexaconazole can be seen clearly embedded in the amorphous phase of chitosan. The absence of hexaconazole crystalline peak in CHEN2.5 might be due to low loading of hexaconazole in the nanoparticles. A sharp peak at diffraction angles (2θ) of 8.4°, 10.5°, 11.7°, 12.2°, 13.9°, 16.0°, 17.0°, 18.3°, 20.2°, 21.1°, 21.7°, 22.0°, 23.4°, 24.0°, 26.1°, 29.4°, 30.9°, 32.0°, and 34.8° matched with the XRD pattern of pure hexaconazole, thus proving the encapsulation of hexaconazole in the chitosan matrix.

#### 2.1.3. FTIR Spectroscopy

**Figure 2:**
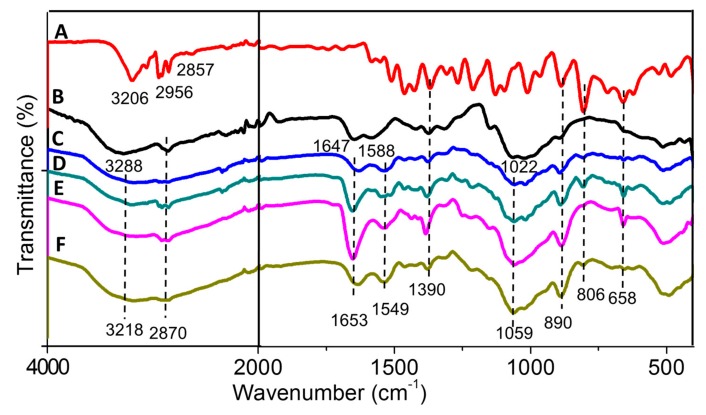
FTIR spectrum of (**A**) pure hexaconazole, (**B**) CEN and chitosan–hexaconazole nanoparticles prepared at various concentrations of TPP, (**C**) 2.5, (**D**) 5, (**E**) 10, and (**F**) 20 mg/mL.

As shown in Figure 2, broad bands at 3288 and 1647 cm^−1^ were due to the enhanced hydrogen bonding and electrostatic interaction of the chitosan amino group and the TPP phosphate group in chitosan–TPP nanoparticles, respectively. The band at 1022 cm^−1^ was due to the phosphate group of TPP [38]. Moreover, a band at 3206 cm^−1^ corresponded to the OH group of hexaconazole [39]. Therefore, a band at 3218 cm^−1^ of the synthesized nanoparticles CHEN2.5, CHEN5, CHEN10, and CHEN20 was due to the combination of bands of hydrogen bonding of the chitosan–TPP and hexaconazole. Furthermore, chitosan showed characteristic broad bands at 1647 and 1588 cm^−1^ which indicated the stretching of the CO–NH_2_ group and NH_2_ group bending vibration, respectively [38]. All the synthesized nanoparticles showed the chitosan–TPP characteristic bands with a slight shifting of the bands at 1653, 1549, 1059 cm^−1^. Moreover, additional bands of hexaconazole can be seen for the synthesized nanoparticles at 1390, 890, 806, and 658 cm^-1^ which can be attributed to the C–N stretching, C=C bending, and C–Cl stretching, respectively [39], thus proving the encapsulation of hexaconazole into the chitosan matrix.

#### 2.1.4. Thermal Analysis

**Figure 3:**
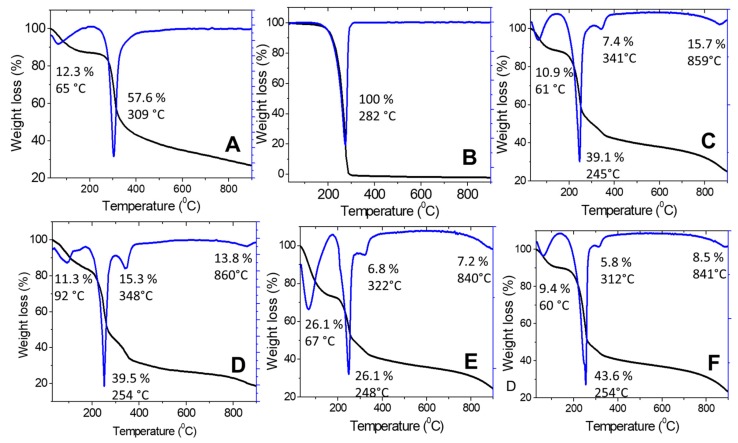
TGA/DTG thermograms of (**A**) CEN, (**B**) pure hexaconazole and chitosan–hexaconazole nanoparticles prepared at various concentrations of TPP, (**C**) 2.5, (**D**) 5, (E) 10, and (**F**) 20 mg/mL.

Thermal stability of the synthesized nanoparticles was studied using a thermal analyzer, and the TGA/DTG thermograms and the data obtained are shown in Figure 3. The results provided quantitative information about the components in the synthesized chitosan–hexaconazole nanoparticles. CEN showed two stages of weight loss: at 65 °C for release of water molecules and 309 °C for the decomposition of chitosan by losing hydrogen bonding. In addition, at the end of the analysis, nearly 27% of the sample remained as residue, indicating higher thermal stability of chitosan. Moreover, 100% weight loss was obtained at 282 °C for pure hexaconazole, which indicated a total decomposition of hexaconazole.

Moreover, the synthesized nanoparticles of CHEN2.5, CHEN5, CHEN10, and CHEN20 showed similar patterns with four stages of weight loss. The first stage of weight loss at around 60 °C (for CHEN5, CHEN10, and CHEN20) and 92 °C for CHEN5, was due to the release of water molecules. The second stage at 245–255 °C was attributed to the decomposition of chitosan, while the third stage at 332–352 °C was due to the decomposition of hexaconazole, thus showing higher thermal stability of hexaconazole in the CHEN2.5, CHEN5, CHEN10, and CHEN20 nanoparticles compared to their pure hexaconazole. For the last stage at around 890 °C, the weight loss was attributed to the char due to the decomposition of chitosan.

#### 2.1.5. Morphology and Particle Size Distribution

**Figure 4:**
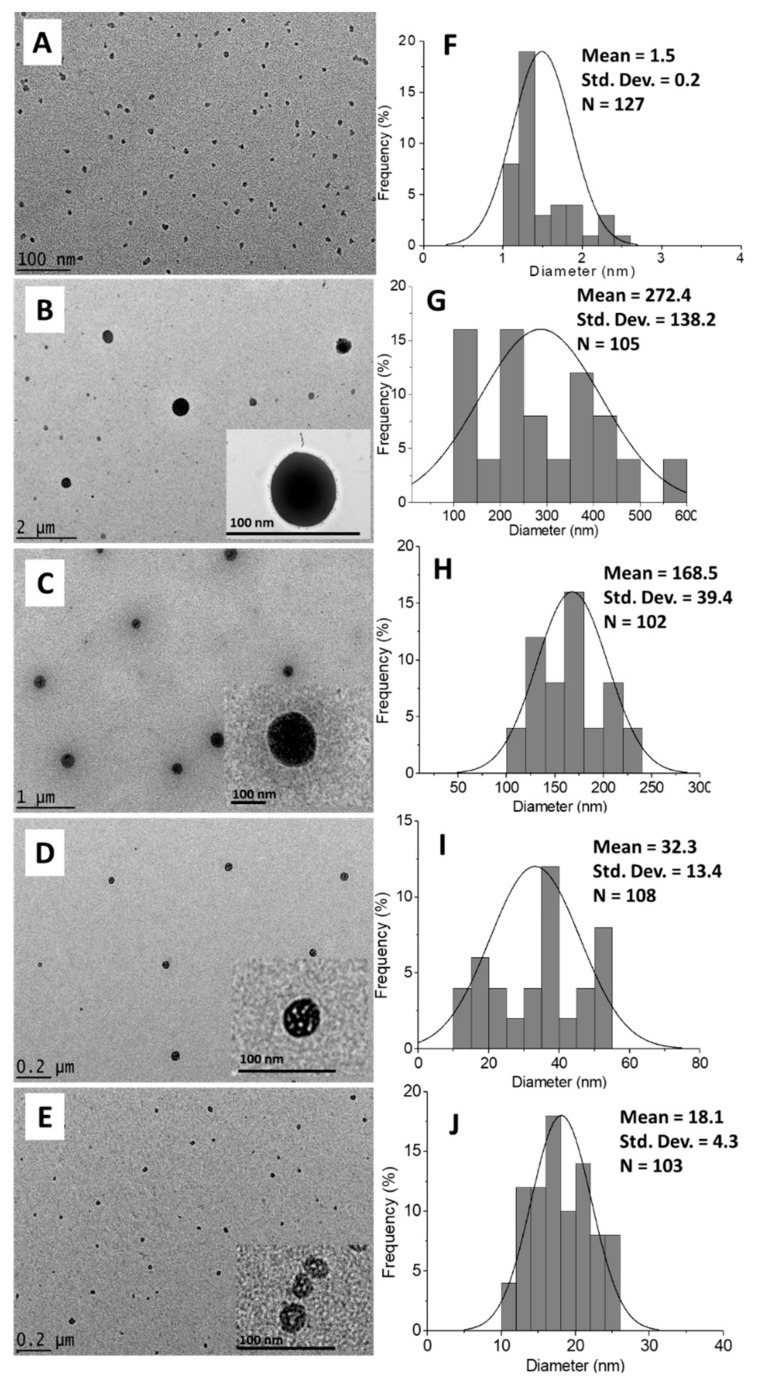
HRTEM image of (**A**) CEN and chitosan–hexaconazole nanoparticles prepared at various concentrations of TPP, (**B**) 2.5, (**C**) 5, (**D**) 10, and (**E**) 20 mg/mL and their particle size distribution of (**F**) CEN and chitosan–hexaconazole nanoparticles prepared at various concentrations of TPP, (**G**) 2.5, (**H**) 5, (**I**) 10, and (**J**) 20 mg/mL.

The morphology of CEN and synthesized chitosan–hexaconazole nanoparticles CHEN2.5, CHEN5, CHEN10, and CHEN20 were studied by HRTEM (Figure 4A–E). Meanwhile, their particle size distribution was measured via ImageJ software (Figure 4F–J). As shown in Figure 4, a sphere shape was obtained for both unloaded CEN and hexaconazole-loaded chitosan, CHEN2.5, CHEN5, CHEN10, and CHEN20. The particle size distribution showed a lower range of size was obtained for the CEN with the mean size diameter of 1.5 nm. The addition of hexaconazole resulted in an increase of the mean sphere size, where smaller mean sphere size was obtained with the increase of TPP concentration, with the following trend: CHEN2.5, CHEN5, CHEN10, and CHEN20 with 271.4, 168.5, 32.3, and 18.1 nm, respectively.

**Figure 5:**
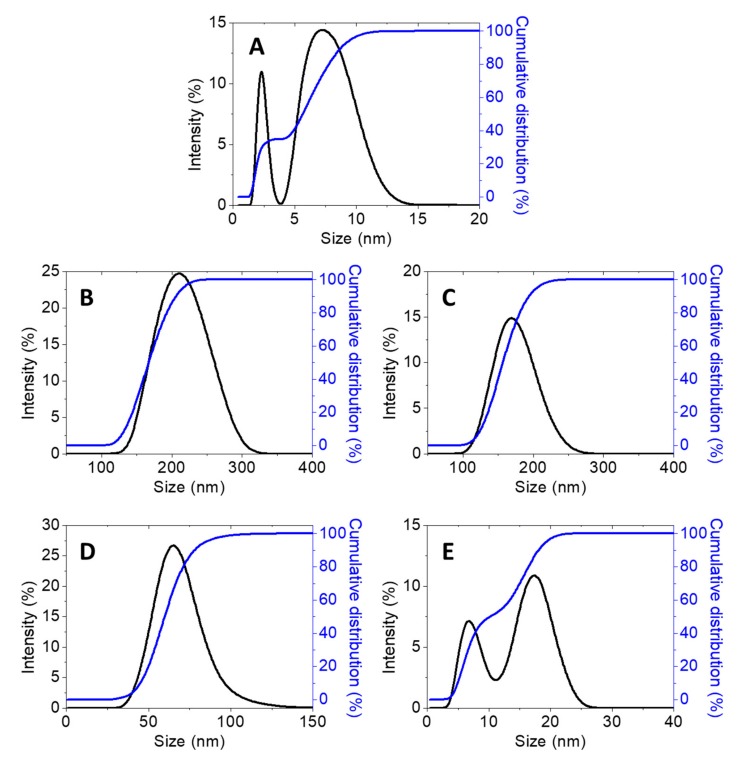
Cumulative and relative particles size distributions (PSD) of (**A**) CEN and chitosan–hexaconazole nanoparticles prepared at various concentrations of TPP, (**B**) 2.5, (**C**) 5, (**D**) 10, and (**E**) 20 mg/mL.

Furthermore, the particle size distribution in the solvated state was done in which there were solvent molecules (deionized water) interacting with the particles. The hydrodynamics size measured via dynamic light scattering (DLS) of the CEN and the synthesized chitosan–hexaconazole nanoparticles CHEN2.5, CHEN5, CHEN10, and CHEN20 are shown in Figure 5. CEN shows a bimodal particle size distribution with peaks at 2.3 and 7.5 nm with 50% PSD of 5.6 nm. This is relatively smaller compared to the size reported in the previous works on chitosan (CS)–TPP nanoparticles. For instance, Kuen et al., Sreekumar et al., Fan et al., and Morris et al. recorded the mean hydrodynamic diameter of CS–TPP was 50–500 nm [30,40,41,42]. As mentioned earlier, TWEEN-80 was added in the current work as a stabilizer. It was reported that TWEEN-80 was able to reduce the surface tension, stabilize the droplet phase, and prevent aggregation in the production of nanoparticles [43,44,45]. This is why the HRTEM image shows that the sphere-like chitosan nanoparticles are seen to be well dispersed.

Moreover, the same pattern was observed where the increase of the TPP concentrations resulted in the decrease of the mean hydrodynamic size. Monomodal particle size distribution was observed for CHEN2.5, CHEN5, and CHEN10 with a peak at 220.2, 164.2, 68.1 nm (50% of PSD; 177.2, 132.1, and 59.2 nm, respectively), while for CHEN 20, it shows a bimodal particle size distribution with peaks at 6.5 and 18.1 nm and 50% PSD of 10.1 nm. The findings of the results also agreed that increasing TPP concentration leads to the decreasing of the particles size. This behavior is presumably due to the increase in the concentration of TPP resulting in the increasing number of negatively charged TPP polyanions, which then facilitate the crosslinking with the positively charged functional groups of the chitosan. This is because, under acid condition, the –NH_2_ functional group of chitosan is protonated to the –NH_3_^+^ [31].

As reported by Chauhan N. et al., it is important to develop a suitable balance between the chitosan and TPP in order to produce the particles in the nanometer range [46]. They also reported that a further increase of TPP concentration may lead to enhancement of the nanocapsule of chitosan–hexaconazole size, which contradicts our finding in this present study. Hence, a TPP-to-chitosan ratio of 1:2.5 (*v*/*v*) is believed to be a suitable ratio in the preparation of chitosan–hexaconazole nanoparticles.

### 2.2. In Vitro Hexaconazole Release

**Figure 6:**
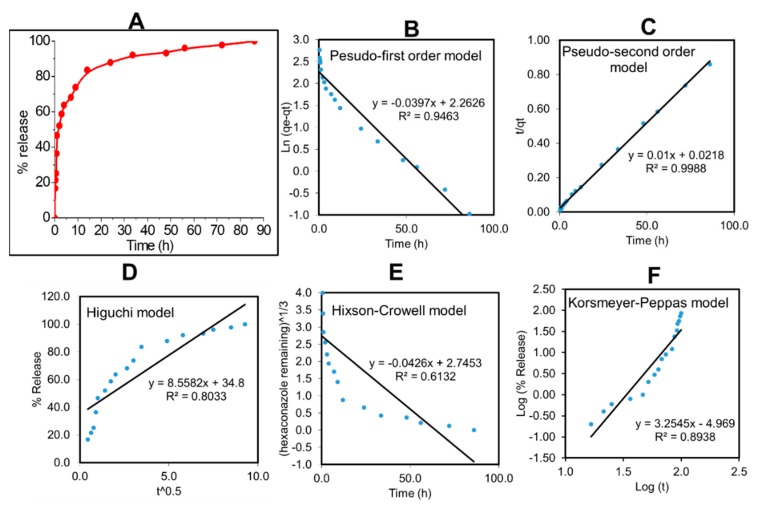
Cumulative release profiles of the (**A**) CHEN5 at pH 5.5 and (**B–F**) their fitting of the data using five different mathematical models at pH 5.5.

To investigate the delivery of hexaconazole in response to time, CHEN5 was incubated in a phosphate buffer saline solution at pH 5.5. CHEN5 was chosen for this study due to its highest loading of hexaconazole compared to the others. As shown in Figure 6, CHEN5 showed a small burst effect in the first 6 hours. Then, a sustained release of hexaconazole in CHEN5 was achieved for up to 86 hours with 99.91% release. The small burst effect release of hexaconazole may be attributed to the hexaconazole located close to the surface of the sphere of CHEN5 nanoparticles. 

To develop an effective fungicide nanodelivery system, it is crucial to determine the fungicide release profiles using model-dependent methods including the pseudo-first-order and pseudo-second-order kinetics and mathematical models including Higuchi, Hixson–Crowell, and Korsmeyer–Peppas models. By fitting the hexaconazole release data into five different kinetic models, the linear fits of curves of different release behaviors are presented in Figure 6 and Table 2.

The linear forms in the first-order kinetic model and second-order kinetic model are shown in Equations 1 and 2, respectively, where K_1_ and K_2_ are the rate constants for the pseudo-first-order and pseudo-second-order release kinetics, respectively. q_e_ and q_t_ represent the quantities of hexaconazole released at equilibrium and at any time (t), respectively. The Higuchi model (Equation 3) describes the hexaconazole release from the nanoparticles with the square root of time, where K_H_ is the Higuchi rate constant. The Hixson–Crowell model (Equation 4) reveals a relationship between the cube root hexaconazole remaining in the nanoparticles as a function of time, where K_HC_ is the Hixson–Crowell rate constant, M_o_ is the initial quantity of the hexaconazole in the nanoparticles, and q_t_ is the quantity released at time t. The Korsmeyer–Peppas (Equation 5) model describes a relationship between the log of the hexaconazole release percentage and the log of time, where q_∞_ is the release at the infinite time and n is the release exponent.

Ln (q_e_ − q_t_) = ln q_e_ − K_1_ t (1)

t/q_t_ = 1/K_2_q^2^_e_ + t/q_e_ (2)

q_t_ = K_H_ √t (3)

^3^√M_0_ − ^3^√q_t_ = K_HC_t (4)

qt/q∞ = Kt^n^ (5)

The calculated correlation coefficient (R^2^) and rate constant (K) values (Table 2) of the hexaconazole release data reveal that the release kinetics of CHEN5 fitted better to the pseudo-second-order kinetic compared to the other models. This indicates that the overall reactions are dependent upon the ion exchange between the hexaconazole molecules and the release medium at the time of release and at the equilibrium with the t_1/2_ of 41.97 hours [47,48].

### 2.3. In Vitro Antifungal Activity Assay on G. boninense

*In vitro* antifungal evaluations were done in several conditions: a control, where the mycelia was plated on PDA with solvent only; the host, CEN, pure hexaconazole; and the synthesized nanoparticles, CHEN2.5, CHEN5, CHEN10, and CHEN20. Their inhibitory effect on *G. boninense* was evaluated based on the inhibition rate and the calculated EC_50_ value, with a higher inhibition rate showing better antifungal activity against *G. boninense*. On the contrary, the lower the EC_50_ value, the more effective the fungicide was in killing the *G. boninense*.

**Figure 7:**
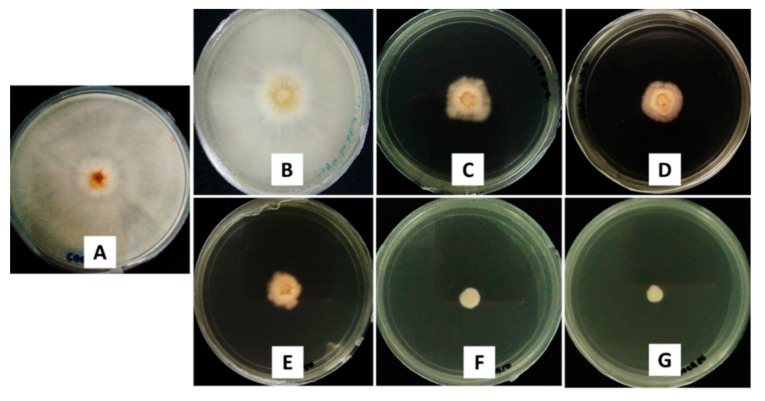
Antifungal effect on *G. boninense* of the (**A**) control, 50 ppb of (**B**) CEN, (**C**) pure hexaconazole and chitosan–hexaconazole nanoparticles prepared at various concentrations of TPP, (**D**) 2.5, (**E**) 5, (**F**) 10, and (**G**) 20 mg/mL, seven days after incubation at 28 ± 2 °C.

The antifungal activity was analyzed using the mycelia growth method. As shown in Figure 7, at a concentration of 50 ppb, similar to the control, CEN showed no inhibitory effect, as the maximum mycelial growth was achieved (radius of 40.00 mm). In addition, the significant inhibitory effect can be seen for the pure hexaconazole and synthesized nanoparticles, CHEN2.5, CHEN5, CHEN10, and CHEN20, as the mycelial growth was much smaller. Pure hexaconazole showed a mycelial growth at a radius of 5.75 mm. Interestingly, as the concentration of TPP increased, the size of the nanoparticles became smaller, resulting in a smaller radius of the mycelial mean growth: 5.25, 2.02, 1.33, and 0.25 mm for CHEN2.5, CHEN5, CHEN10, and CHEN20, respectively.

**Figure 8:**
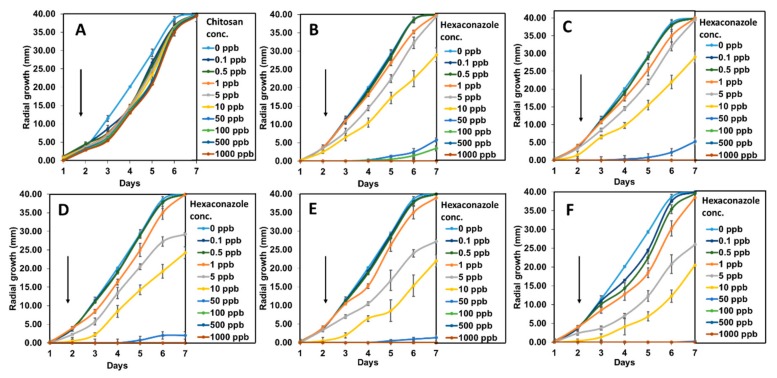
*G. boninense* growth curve from day 1 to 7 incubated in (**A**) CEN, (**B**) pure hexaconazole and chitosan–hexaconazole nanoparticles prepared at various concentrations of TPP, (**C**) 2.5, (**D**) 5, (**E**) 10, and (**F**) 20 mg/mL at 28 ± 2 °C and at increasing concentration of 0–1000 ppb. Black arrows represent the increasing of the hexaconazole concentration and the error bars represent the standard deviation of the mean.

To get a better understanding, the growth curves of *G. boninense* incubated in modified PDA with aqueous pure hexaconazole, CEN, and synthesized nanoparticles were plotted as shown in Figure 8. The inhibitory effect of CEN was almost negligible as the mycelial growth was almost similar to the control. Pure hexaconazole showed significant inhibitory effect starting from 100 ppb with zero radii mycelial mean growth at 500 ppb. Moreover, the enhanced inhibitory effect can be seen clearly for the synthesized chitosan–hexaconazole nanoparticles at various concentrations of TPP.

**Figure 9:**
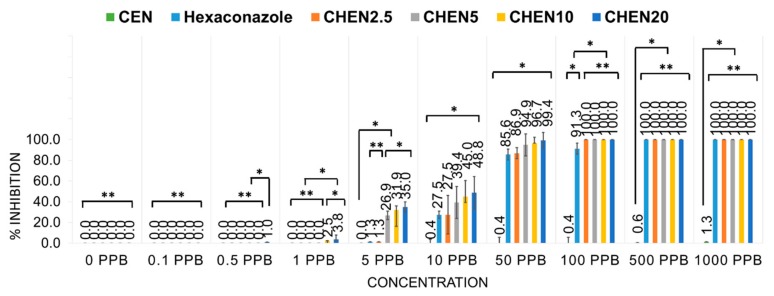
Percentage inhibition of radial growth of *G. boninense* against concentration, seven days after incubation at 28 ± 2 °C; where * *p* < 0.01 (significant) and ** *p* > 0.5 (not significant); the error bars represent the standard deviation of the mean.

In addition, the calculated percentage inhibition of mycelial mean radial growth of *G. boninense* is presented in Figure 9. At 0.5 ppb, only CHEN20 showed inhibition with 1.0%, whereas at 1 ppb, only nanosized CHEN10 and CHEN20 showed the inhibition of *G. boninense* with 2.5 and 3.8%, respectively, which indicates the superior effect of nanosized CHEN10 and CHEN20. At 10 and 50 ppb, the same trend was observed where CEN showed almost negligible inhibition and the synthesized nanoparticles showed higher inhibition compared to their counterpart, pure hexaconazole which follows the order CHEN2.5 < CHEN5 < CHEN10 < CHEN20.

Furthermore, EC_50_ of fungicides was determined using the Sigma Plot 10.0 software as presented in Table 3. CEN showed the highest EC_50_ with a value of 1534.5 ppb, followed by pure hexaconazole with a value of 21.4 ppb. Moreover, the synthesized nanoparticles showed remarkably better antifungal activity on *G. boninense* with the lower EC_50_ of 18.4, 10.8, 9.1, and 8.0 ppb for CHEN2.5, CHEN5, CHEN10, and CHEN20, respectively. From this result, we can conclude that the smaller particle size of chitosan–hexaconazole nanoparticles results in a higher antifungal activity on *G. boninense.*

**Figure 10:**
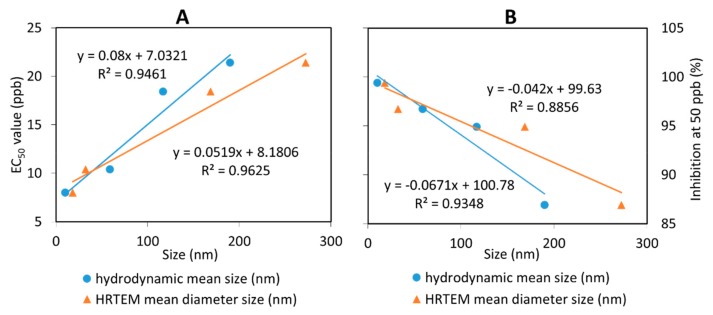
The relationship between the hydrodynamic mean particle size distribution and HRTEM mean particle size distribution of the synthesized chitosan–hexaconazole nanoparticles to (**A**) their percentage inhibition at 50 ppb and (**B**) the calculated EC_50_ (ppb) value on *G. boninense*.

In order to study the relationship between particle size of the synthesized chitosan–hexaconazole nanoparticles and EC_50_ value as well as percentage inhibition of mean mycelial growth of *G. boninense*, the plot of the relationship was done as shown in Figure 10. As discussed earlier, the increase in TPP concentration resulted in the decrease of the particle size. Interestingly, both methods showed that the decrease in particle size is directly proportional to the decrease of lower EC_50_ values (Figure 10A) and also directly proportional to the increase of inhibition percentage (Figure 10B). This revealed that the smaller chitosan–hexaconazole nanoparticles have a higher ability to kill the *G. boninense* which is owing to its larger surface area that can be in contact with the fungus cell [49].

## 3. Materials and Methods

### 3.1. Materials

Hexaconazole (C_14_H_17_Cl_2_N_3_O, the molecular weight of 314.21 g/mol) was purchased from Changzhou Aiteng with 95% purity and was used as received. Chitosan (medium molecular weight, 190,000–310,000 degree of acetylation), acetic acid glacial (100%), TWEEN-80, and sodium tripolyphosphate (TPP) were purchased from Sigma Aldrich (St. Louis, MO, USA). Hydrochloric acid (37%) and *N*,*N*-dimethylformamide (DMF) were purchased from Friedemann Schmidt (Parkwood, Australia) and Merck (Kenilworth, NJ, USA), respectively. All other reagents used were of analytical grade. *G. boninense* culture was provided by Malaysian Palm Oil Board (MPOB), Bangi, Malaysia and maintained in potato dextrose agar (PDA) media from Oxoid, Thermo Scientific (pH 5.5) (Waltham, MA, USA) incubated at 28 ± 2 °C.

### 3.2. Preparation of Chitosan and Its Encapsulation of Hexaconazole Nanoparticles

Chitosan nanoparticles (CEN) were prepared using ionic gelation method [38,50]. Briefly, 0.25 g of chitosan in powder form was dissolved in a 1.0% (*v*/*v*) acetic acid solution and at the same time, 0.2 g of TPP was prepared in 40 mL of deionized water separately. The pH of the resulting mixture was around 3.6. Then, 2% *v*/*v* TWEEN-80 was added as a stabilizer to prevent particle aggregation [43,44]. Sodium TPP solution was added dropwise using a burette into the chitosan solution while stirring. The mixture was then centrifuged at 40,000 rpm for 10 min and the supernatant was discarded. The chitosan nanoparticle pellet was then freeze-dried overnight before further analysis.

Hexaconazole-loaded chitosan nanoparticles (CHEN) were prepared using ionic gelation method [38,50]. Chitosan, 0.5 g, was dissolved in 100 mL of 1.0% (*v*/*v*) acetic acid solution. Due to its low water solubility, 1 g of hexaconazole was dissolved in 100 mL of DMF first and then added to chitosan solution under stirring until a homogenous solution was obtained. Then, 2% *v*/*v* of TWEEN-80 was added as a stabilizer. A series of 40 mL of sodium TPP concentrations of 2.5, 5, 10, and 20 mg/mL (abbreviated as CHEN2.5, CHEN5, CHEN10, and CHEN20, respectively) was prepared in deionized water separately. Next, sodium TPP solution was added dropwise using a burette into the mixture solution while stirring. Final TPP-to-chitosan ratio achieved was 1:2.5 (*v*/*v*). The mixture was then centrifuged at 40,000 rpm for 10 min and the supernatant was discarded. The chitosan–hexaconazole nanoparticles pellet was then freeze-dried overnight before further analysis. CHEN2.5, CHEN5, CHEN10, and CHEN20 were prepared by encapsulating hexaconazole into the chitosan matrix through the crosslinking of electropositive chitosan with electronegative TPP.

### 3.3. Reaction Yield, Hexaconazole Loading Content, and Encapsulation Efficiency

Reaction yield (RY) of the synthesized chitosan–hexaconazole nanoparticles was calculated using Equation 6 [51].

RY = [Produced nanoparticle (mg)/(used chitosan (mg) + used hexaconazole (mg))] × 100(6)

The hexaconazole loading content (LC) and encapsulation efficiency (EE) were determined using the high-performance liquid chromatography (HPLC) technique Briefly, 5.0 mg of the synthesized nanoparticles were dissolved in 10.0 mL methanol and hydrochloric acid (0.5% *v*/*v*) under sonication, and a clear solution was obtained prior to the HPLC analysis. The nanoparticles were ensured to be completely dissolved, thus releasing 100% of hexaconazole content [44]. The mobile phase of HPLC consisted of methanol/water (30:70, *v*/*v*). The flow rate was 1.0 mL/min at 25 °C and the wavelength was set at 235 nm. The retention time obtained was 1.8 min. The hexaconazole LC and EE were calculated according to the following equations:

LC (%) = [weight of hexaconazole in nanoparticles/weight of nanoparticles] × 100(7)

EE (%) = [weight of hexaconazole in nanoparticles/initial amount of hexaconazole in the system] × 100(8)

### 3.4. Characterizations

The structural characterizations of the synthesized nanoparticles were performed via Fourier Transform Infrared spectra (FTIR), powder X-ray diffraction (PXRD), and thermogravimetric and differential thermogravimetric analyses (TGA/DTG) techniques by using the method described earlier [50]. FTIR was performed on a Thermo Nicolet Nexus spectrometer with a Smart Orbit (Waltham, MA, USA) in the range of 400–4000 cm^−1^. PXRD was carried out using Bruker D8 Advance powder XRD (Billerica, MA, USA) using CuK_α_ radiation (λ = 0.15406 nm) at 40 kV and 40 mA. The TGA/DTG analysis was done using Mettler-Toledo 851e (Columbus, OH, USA) at a heating rate of 10 °C min^−1^ in 150 µL alumina crucibles in the range of 30–900 °C.

Prior to the DLS and HRTEM measurement, the nanoparticles were suspended in deionized water and sonicated for 5 minutes. The hydrodynamic particle size distribution was determined by the dynamic light scattering (DLS) method using a particle size analyzer Nano Series Nano-ZS (Malvern Panalytical Ltd., Malvern, United Kingdom). The internal morphology and particle size diameter were studied using HRTEM, FEI Tecnai G2 F20 S-TWIN (Hillsboro, OR, USA).

HPLC assays were performed with a Waters Alliance 2695 separation module (Milford, MA, USA), a diode array detector Waters 2996 at room temperature (23 °C). The C18 column (5µm, 4.6 × 150 mm) with mobile phase 30:70 (*v*/*v*) of methanol:water solution was employed.

### 3.5. Hexaconazole Release Profile Study

Briefly, 30.0 mg of the CHEN pellet collected after centrifugation were dispersed in 30 mL phosphate buffer saline (pH 5.5) and shaken in an incubator shaker (27 °C) at 100 rpm. The solution of pH 5.5 was chosen to imitate the release of the fungicide in the potato dextrose agar, the medium used in the *in vitro* antifungal studies. At predetermined intervals, supernatants were isolated by centrifugation, and 1 mL of the solution was taken out and replaced with the same amount of the fresh medium. The hexaconazole release from the nanoparticles was investigated using high-performance liquid chromatography (HPLC), Waters HPLC system with ELS detector (Shimadzu, Thermo Science, USA). The nonreleased drug that was still entrapped in the nanoparticles was discarded by centrifugation, as the nanoparticles were discarded at the end of the work process. Hence, the supernatant only contained the drug that was released into the medium.

### 3.6. In Vitro Antifungal Assay

The *in vitro* antifungal activity of the synthesized nanoparticles was evaluated against *G. boninense* using the poisoned medium technique, using potato dextrose agar (PDA) medium. The PDA was amended in several different conditions (pure hexaconazole, CEN, CHEN2.5, CHEN5, CHEN10, and CHEN20) at several concentrations (0.1, 0.5, 1, 5, 10, 50, 100, 500, 1000 ppb of the active ingredient), which were prepared in 0.5% HCl. Medium with only solvent served as a control. A 5 mm portion of the mycelial disc from margins of actively growing culture of *G. boninense* was placed at the center of amended PDA. The radial growth of the mycelia was measured for seven days of inoculation by incubating the petri plates at 28 ± 2 °C (*n* = 5). Mycelial growth was recorded every day. The percentage inhibition of the radial growth by the fungicide was then calculated.

### 3.7. Statistical Analysis

Data are presented as mean ± standard deviation and the statistical difference of the parameters was analyzed using the ANOVA and Tukey’s test (*p* ≤ 0.05) using the SPSS software. The half maximum effective concentration (EC_50_) of chitosan–hexaconazole nanoparticles was determined using the sigma plot analysis of Sigma Plot 10.0.

## 4. Conclusions

In summary, various sizes of chitosan–hexaconazole nanoparticles managed to be synthesized using the ionic gelation method using sodium tripolyphosphate (TPP) of various concentrations: 2.5, 5, 10, and 20 mg/mL. HRTEM revealed that the chitosan nanoparticles were in a sphere shape, while the particle size of chitosan–hexaconazole nanoparticles was found to decrease due to the increased concentration of TPP. In addition, the chitosan–hexaconazole nanoparticles for all TPP concentrations exhibited better thermal stability compared to hexaconazole. Meanwhile, chitosan–hexaconazole nanoparticles at 5 mg/mL of TPP presented the highest loading content of 16.7% as well as the sustained release of 99.91% with a prolonged release time of hexaconazole up to 86 hours. Apart from that, the *in vitro* antifungal assay revealed that smaller particle size of the chitosan–hexaconazole nanoparticles provided better inhibition of *Ganoderma boninense* with lower EC_50_ values with the following order: CHEN2.5 > CHEN5 > CHEN10 > CHEN20 with 18.4, 10.8, 9.1, and 8.0 ppb, respectively. Most importantly, the proposed fungicide nanodelivery system provided longer efficient time, low toxicity, and high antifungal activity towards *G. boninense* as well as low EC_50_ value, thus making it a promising candidate in combating and treating *Ganoderma* disease in oil palm.

## Figures and Tables

**Table 1:** Reaction yield, loading content, and encapsulation efficiency of the synthesized nanoparticles.

Synthesized Nanoparticles	Reaction Yield * (%)	Loading Content * (%)	Encapsulation Efficiency * (%)
**CHEN2.5**	65.5 ± 3.5 ^a^	10.7 ± 2.2 ^a^	55.7 ± 4.3 ^a^
**CHEN5**	75.0 ± 4.0 ^b^	16.7 ± 3.5 ^b^	66.7 ± 1.5 ^b^
**CHEN10**	74.5 ± 2.5 ^b^	15.4 ± 2.5 ^b^	65.4 ± 2.0 ^b^
**CHEN20**	76.0 ± 2.5 ^b^	15.2 ± 3.0 ^b^	65.3 ± 3.5 ^b^

* Different letters (^a,b^) in the same column indicate significant differences between means (*p* ≤ 0.05) according to Tukey’s test.

**Table 2:** The correlation coefficients (R^2^) and rate constant (K) obtained by fitting the hexaconazole release data from the CHEN5 in PBS solution pH 5.5.

Sample	Saturation Release (%)	Pseudo-First-Order		Higuchi Model		Pseudo-Second-Order		
CHEN5	99.91	R^2^	K_1_ (ln mg h^−1^)	R^2^	K_H_ (mg √h^−1^)	R^2^	K_2_ (mg h^−1^)	t_1/2_ (h)
		0.9463	−0.0397	0.8033	8.5582	0.9988	0.0100	41.97
		**Hixson–Crowell Model**		**Korsmeyer–Peppas Model**				
		R^2^	K_HC_ (h^−1^)	R^2^	K (h^−1^)			
		0.6132	−0.0426	0.8938	3.2545			

**Table 3:** Calculated EC_50_ value of CEN, pure hexaconazole, and chitosan–hexaconazole nanoparticles prepared at various concentrations of TPP on *G. boninense* at day 7 of incubation at 28 ± 2 °C.

Parameter	Type of Fungicides					
	CEN	Hexaconazole	CHEN2.5	CHEN5	CHEN10	CHEN20
EC_50_ (ppb)	1534.5	21.4	18.4	10.8	9.1	8.0
Fiducial limit (ppb) (lower-upper)	494.0–13280.4	16.7–27.3	13.0–32.8	8.1–16.3	6.8–12.9	6.0–10.9
